# Regulatory SNP of *RREB1* is Associated With Bone Mineral Density in Chinese Postmenopausal Osteoporosis Patients

**DOI:** 10.3389/fgene.2021.756957

**Published:** 2021-11-18

**Authors:** Shuo Feng, Han Wang, Yumeng Yan, Xin Su, Jintao Ao, Wei Chen

**Affiliations:** ^1^ Department of Spine Surgery, Beijing Jishuitan Hospital, Beijing, China; ^2^ Key Laboratory for Biomechanics and Mechanobiology, Ministry of Education, Beijing Advanced Innovation Centre for Biomedical Engineering, School of Biological Science and Medical Engineering, Beihang University, Beijing, China; ^3^ Beijing GuardianHealth Technology Co., Ltd., Beijing, China

**Keywords:** bone mineral density, postmenopausal osteoporosis, genome-wide association study, *RREB1*, splicing quantitative trait locus

## Abstract

Postmenopausal osteoporosis (PMO) is the most common bone disorder in elderly Chinese women. Although genetic factors have been shown to have a pivotal role in PMO, studies on genetic loci associated with PMO in Chinese individuals are still lacking. We aimed to identify SNPs that contribute to PMO in Chinese individuals by conducting a genome-wide association study (GWAS). Bone mineral density (BMD) of postmenopausal Chinese women was assessed. Participants with T-score < −2.5 standard deviations (*n* = 341) were recruited and divided into a discovery group (*n* = 150) and a replication group (*n* = 191). GWAS was performed, with T-score as the quantitative trait, using linear regression. Our results revealed that an SNP cluster upstream of *RREB1* showed a trend of association with BMD in Chinese PMO patients. The leading SNP of the cluster was rs475011 (*p*_combined_ = 1.15 × 10^−6^, beta = 0.51), which is a splicing quantitative trait locus (sQTL) of *RREB1*. This association was further supported by data from the UK Biobank (UKBB; *p* = 9.56 × 10^−12^). The high BMD-associated allele G of rs475011 is related to a high intron excision ratio. This SNP may increase BMD by upregulating mature *RREB1* mRNA, based on data from the Genotype-Tissue Expression (GTEx) database. We identified BMD-associated SNPs that regulate *RREB1* in Chinese PMO patients. Future functional experiments are needed to further link rs475011, *RREB1*, and PMO in Chinese individuals.

## Introduction

Postmenopausal osteoporosis (PMO) is the most common bone disorder in elderly women worldwide (Black and Rosen, 2016; Eastell et al., 2016). In China, its estimated prevalence is 34.65% (Chen et al., 2016). It is characterized by altered bone microstructure and decreased bone mineral density (BMD), and it is the leading cause of nonstress fractures in the elderly (Zhuang et al., 2020; Porter and Varacallo, 2021). The risk factors for PMO include decreased postmenopausal estrogen levels, smoking, decreased physical activity, low body mass index (BMI), and genetic susceptibility (Eastell et al., 2016; Bijelic et al., 2017). The heritability of BMD is 0.6–0.8 (Peacock et al., 2002) and the heritability of osteoporotic fracture is 0.5–0.7 (Deng et al., 2002). These findings indicate that genetics play a pivotal role in PMO.

Genome-wide association studies (GWASs) are useful for identifying genetic susceptibility factors underlying diseases and phenotypes (Visscher et al., 2017). Early single-sample GWASs identified associations of SNPs in *RANKL*, *OPG*, *ESR1,* and *TNFRSF11B* with BMD in individuals of European descent (Richards et al., 2008; Styrkarsdottir et al., 2008). By integrating data from multiple studies, the GEnetic Factors for OSteoporosis Consortium (GEFOS) conducted a GWAS meta-analysis of >580,000 individuals. They identified 15 loci associated with BMD and fractures, including SNPs in *SOST*, *WNT16*, and *ESR1* (Trajanoska et al., 2018). The susceptibility SNPs identified in the GEFOS study explain 6–10% of the phenotypic variance in BMD (Medina-Gomez et al., 2018; Yang et al., 2020). Furthermore, data on ∼420,000 individuals from the UK Biobank (UKBB) were used to link 518 genetic loci with BMD, explaining 20% of the phenotypic variance (Morris et al., 2019). The susceptibility genes are related to osteoblast and osteoclast differentiation, which further supports the importance of genetic factors in PMO (Yang et al., 2020).

The majority of the genetic susceptibility loci regarding BMD and osteoporosis have been obtained from individuals of European descent. There is a lack of GWAS data from Chinese populations. Kung et al. reported an association between rs2273061 of *JAG1* with BMD among 800 Han Chinese women using a case-control GWAS (Kung et al., 2010). Guo et al. linked rs13182402 of *ALDH7A* with osteoporotic fractures in 700 elderly Han Chinese subjects (Guo et al., 2010). Tan et al. identified SNPs upstream of *ATP6V1G1* that were associated with both age at menarche and BMD in 800 Chinese participants, using bivariate GWAS (Tan et al., 2015). The fact that the susceptibility loci identified in Chinese subjects exhibit little overlap with those identified in European subjects indicates differences in genetic risk factors for PMO between Chinese and European populations. More research on the genetic contributors to PMO in Chinese individuals is crucial.

We aimed to conduct a GWAS using the T-score (which is a critical measurement of BMD and osteoporosis) as the quantitative trait in Han Chinese PMO patients to identify genetic factors influencing the severity of the disease. We found that SNPs upstream of *RREB1* were significantly associated with BMD. This finding provides new information on the genetic basis of PMO in Chinese individuals.

## Materials and Methods

### Ethics Statement

This study was conducted according to the principles of the Declaration of Helsinki and approved by the ethics committees of Beijing Jishuitan Hospital. All of the patients provided written informed consent, and the ethics committees approved the consent procedure.

### Study Subjects

A total of 341 unrelated Han Chinese postmenopausal women aged >54 years were recruited from Beijing Jishuitan Hospital. They were diagnosed with PMO using World Health Organization criteria (WHO Scientific Group on the Prevention and Management of Osteoporosis, 2003). In brief, the lumbar vertebra BMD of each participant was measured by dual-energy X-ray absorptiometry (DXA; Prodigy; GE Healthcare, Boston, MA) or quantitative computed tomography (qCT; uCT710; United-imaging, Shanghai, China). T-score was obtained by comparison with the BMD of healthy young women. Patients with a T-score less than −2.5 SD were diagnosed with PMO. Data on PMO-related characteristics and environmental exposures (such as BMI, age of menopause, age of menarche, hypertension, type 2 diabetes, and smoking and drinking habits) were also collected. No participants had other diseases that may affect bone mass or were taking hormone replacement therapy.

Pearson’s correlation test was adopted to test the relationship between pairs of all collected phenotypes and environmental exposures in our samples. To calculate the correlation coefficient between binary traits and quantitative traits, binary traits or exposures such as hypertension, type 2 diabetes (T2D), and smoking and drinking habits were coded as 1 for affected or exposed and 0 for unaffected or unexposed. The relationship between BMD and hypertension or T2D was further tested by Student’s t-test. The statistical analysis was performed on R 4.0 software.

### DNA Extraction and Genotyping

A whole-blood sample was obtained from each participant using an ethylenediaminetetraacetic acid (EDTA) blood collection tube (BD, Franklin Lakes, NJ). The DNA was extracted using a QIAamp DNA Mini Kit (Qiagen, Hilden, Germany) according to the manufacturer’s protocol. The blood samples and extracted DNA were stored at -80 °C prior to genotyping.

In the discovery stage of GWAS, whole-genome genotyping was performed using an Illumina Infinium Asian Screening Array (Illumina, San Diego, CA) according to the manufacturer’s protocol. Data management and experimental quality control were conducted using GenomeStudio 2.0 software (Illumina, San Diego, CA).

In the replication stage of GWAS, genotyping of candidate SNPs (selected based on the results of the discovery stage) was conducted using a MassARRAY system (Agena Bioscience, San Diego, CA) according to the manufacturer’s protocol. The genotyping experiments were designed using online Agena tools (https://agenacx.com). Data management and experimental quality control were conducted using Typer 4.0 software (Agena Bioscience, San Diego, CA).

### Discovery Stage

In the discovery stage of GWAS, whole-genome genotyping data were analyzed using a PLINK 1.07 software suite (Purcell et al., 2007). First, stringent filtering was conducted; individuals with genotyping call rate <95%, inbreeding coefficient >0.025, or pairwise identity-by-descent (IBD) > 0.05 were excluded. SNP loci with call rate <90%, minor allele frequency (MAF) < 5%, or significant deviation from Hardy–Weinberg equilibrium (HWE, *p* < 0.01) were also removed. Single-allele association analysis was performed by comparing SNP allele counts with T-score using linear regression, with age and BMI as covariates. Since T-score is a measurement of standard normalized BMD, we used this value as a quantitative trait for association directly (Lewiecki and Lane, 2008). The genome-wide trend of association threshold was set at *p* < 10^−5^.

### Replication Stage

In the replication stage, the leading SNPs of each of the BMD-associated SNP clusters and 1 additional SNP in the SNP cluster upstream of *RREB1* (which was the second leading SNP in this cluster) were selected for validation. Linear regression was used to verify the associations between candidate SNP allele counts and T-score. Age and BMI were used as the covariates. The significance threshold was set at *p* < 0.05.

To replicate our result on public data, summary statistics of GWAS results of heel T-score were downloaded from the UK Biobank (UKBB) pan-ancestry genetic analysis portal (https://pan.ukbb.broadinstitute.org). The selected phenotype was “Heel bone mineral density (BMD) T-score, automated”.

### Meta-Analysis

The final association results of our samples were obtained by combining the results from the discovery and replication stages using the meta-analysis module of PLINK 1.07 (Purcell et al., 2007), using a random- or fixed-effects model for loci with Cochrane’s Q statistic <0.05 or >0.05, respectively. The meta-analysis between our data and UKBB data was also performed by PLINK 1.07.

## Results

### Participant Grouping and Characteristics

**FIGURE 1:**
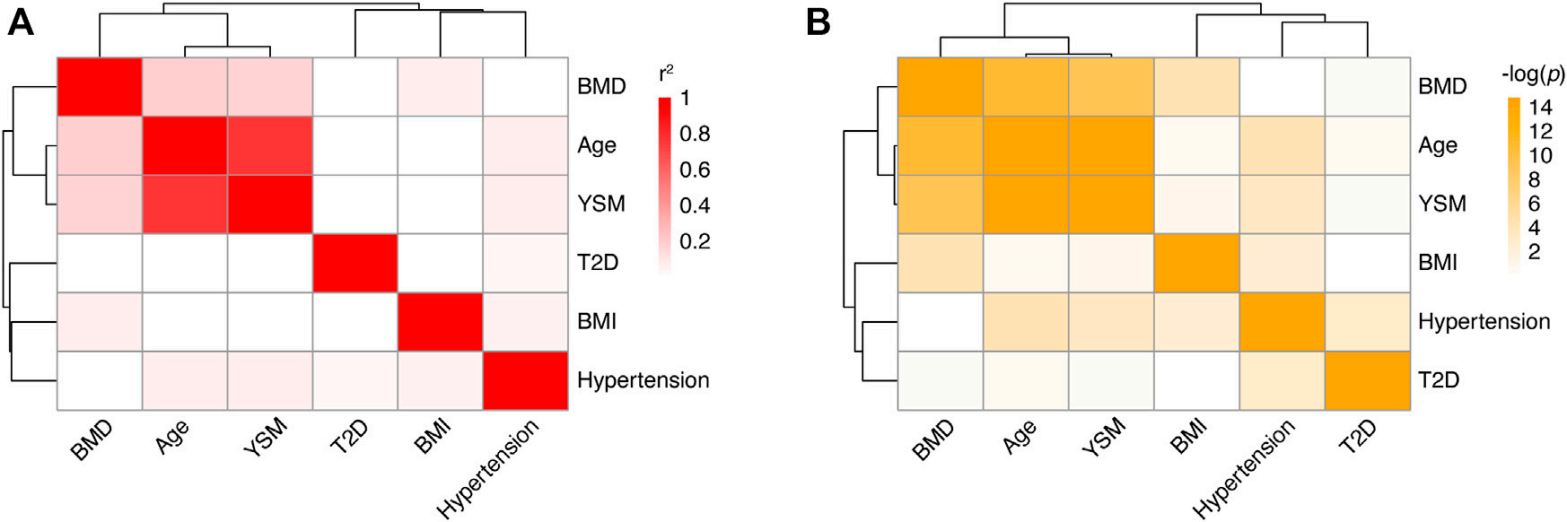
Correlations among different phenotypes in PMO patients. **(A)** Heatmap of correlation coefficients between pairs of phenotypes. Each box represents the Pearson’s *r* ^2^ between the column and row phenotypes. The shade of red indicates the value of *r* ^2^. **(B)** Heatmap of correlation *p* values between pairs of phenotypes. Each box represents the log-transformed *p* value of the correlation between the column and row phenotypes. BMD: bone mineral density; YSM: years since menopause; BMI: body mass index; T2D; type 2 diabetes.

In total, 341 PMO patients were recruited. All the patients were Han Chinese and diagnosed with PMO using DXA or qCT, based on T-score less than -2.5 SD. They were divided into two groups: 150 were used in the discovery stage of GWAS and 191 in the replication stage. The majority of samples in the discovery stage were collected during 2019–2020, and samples in the replication stage were collected during 2020–2021. Data on age, age of menopause, age of menarche, BMI, BMD, and T-score were collected (Table 1). Data on whether the participants had hypertension or T2D were also collected (Table 1). By analyzing the relationships among these variables in our samples (Figure 1 and Supplementary Table S1), we found that age (*p* = 1.25 × 10^−11^, Pearson’s r = −0.44) and years since menopause (YSM, *p* = 3.83 × 10^−10^, Pearson’s r = −0.41) were significantly negatively correlated with BMD, and BMI was significantly positively correlated with BMD (*p* = 4.85 × 10^−5^, Pearson’s r = 0.27), which is consistent with previous reports (Ravn et al., 1999; Warming et al., 2002). However, hypertension (*p* = 0.73, t-test) and T2D (*p* = 0.67, t-test) did not affect BMD. As only ∼70% of the participants had YSM data available (Table 1) and there was a high correlation between age and YSM (*p* < 10^−16^, Pearson’s r = 0.87), we finally used age and BMI as covariates in the GWAS.

**TABLE 1:** PMO-related characteristics of the participants.

	Discovery group	Replication group
Sample size	150	191
Age	72.04 ± 8.03	65.19 ± 6.84
Age of menarche	14.27 ± 1.08 (70.4%^a^)	14.27 ± 1.17 (72.6%^a^)
Age of menopause	48.37 ± 3.13 (72.4%^a^)	49.28 ± 3.81 (67.0%^a^)
Years since menopause	22.87 ± 8.84 (72.4%^a^)	15.53 ± 7.29 (67.0%^a^)
BMI	25.16 ± 3.67	25.45 ± 3.36
BMD	54.03 ± 31.86	73.03 ± 29.08
T-score	-3.89 ± 1.07	-3.60 ± 1.07
Hypertension, n (%)	80 (53.3%)	81 (42.4%)
Type 2 diabetes, n (%)	34 (22.7%)	34 (17.8%)
Smoking, n (%)	1 (0.7%)	6 (3.1%)
Drinking, n (%)	0 (0%)	3 (1.6%)

a Percentage of participants with available data on this phenotype.

### The Discovery Stage of GWAS Identified Loci Associated With T-Score in PMO Patients

To assess the contribution of genetic polymorphisms to BMD in PMO patients, we conducted a GWAS using T-score as the quantitative trait. In the discovery stage, genome-wide genotyping was performed on 150 patients (Table 1) using an Illumina Infinium Asian Screening Array. In total, 705,335 autosomal SNPs were genotyped. As part of the stringent quality control process, SNPs with missing rate >10%, MAF <5%, or significant deviation from HWE (*p* < 0.01) were removed from further analysis, leaving 311,319 loci. We also performed patient-level quality control, which involved identifying individuals with call rate <95%, inbreeding coefficient >0.025, or pairwise IBD >0.05. No genotyped patients met these thresholds, and so all of them were included in the GWAS.

**FIGURE 2:**
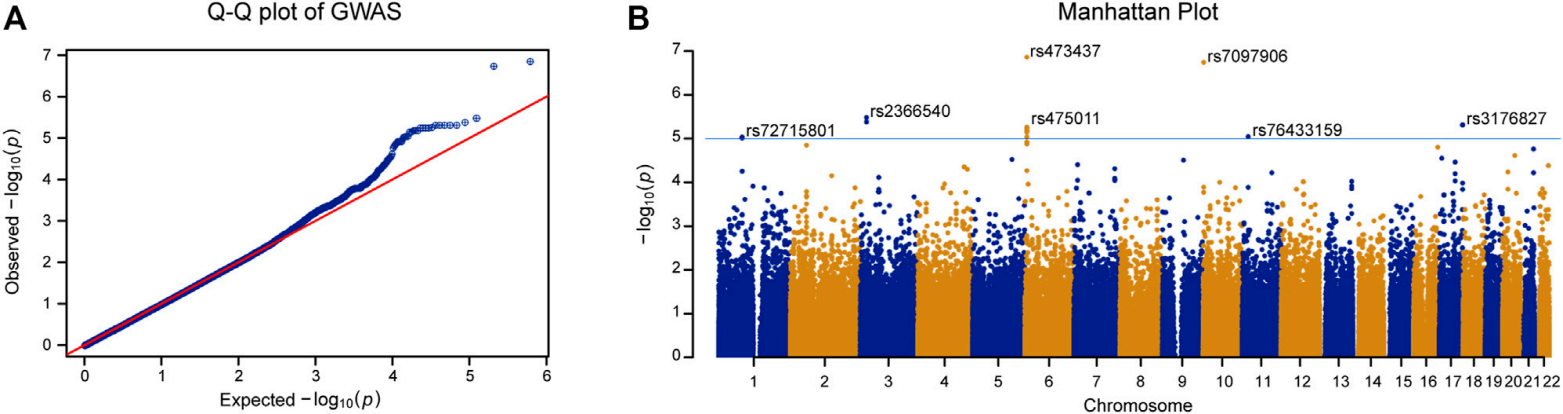
GWAS of T-score in PMO patients. **(A)** Quantile-quantile plot of the GWAS *p* value. X-axis represents the expected *p* value. Y-axis represents the observed *p* value based on linear regression. Red line shows the theoretical distribution under the null hypothesis of no association in the data. **(B)** Manhattan plot of GWAS results. X-axis represents physical position of each tested SNP. Y-axis indicates log-transformed *p* value. Blue line shows the trend of association threshold of 10^−5^. The names of leading SNPs in each associated SNP cluster are shown in the panel.

We carried out linear regression with age and BMI as covariates to assess the associations between genotypes and T-score. As shown in Figure 2A, the quantile-quantile plot of the genome-wide *p* value exhibited significant deviation from the null distribution at the tail of the distribution. The genomic inflation factor was calculated as 1.014. This indicates little population stratification and the existence of genetic factors related to BMD in the PMO patients. Using a lenient threshold of *p* < 10^−5^ as a trend of genome-wide association, we identified 6 SNP clusters associated with BMD in the PMO patients (Figure 2B; Table 2 and Supplementary Table S2).

**TABLE 2:** SNPs showed trend of association with BMD in PMO patients.

SNP	Gene	Chr	Minor allele	Discovery group		Replication group		Combined		
				Beta (95%CI)	*p*	Beta (95%CI)	*p*	Beta^a^	P_combined_ ^a^	Q
rs473437	*RREB1*	6	T	0.84 (0.54, 1.13)	1.40 × 10^−7^	0.32 (0.03, 0.61)	0.03305	0.58	0.02	0.01
rs7097906	—	10	G	0.92 (0.59, 1.25)	1.83 × 10^−7^	0.13 (-0.16, 0.41)	0.3875	0.52	0.19	0.00
rs2366540	*SGO1-AS1*	3	C	1.06 (0.63, 1.50)	4.14 × 10^−6^	-0.24 (-0.78, 0.30)	0.3783	0.42	0.52	0.00
rs3176827	*SLC16A3*	17	A	1.17 (0.69, 1.65)	4.84 × 10^−6^	-0.10 (-0.45, 0.25)	0.5806	0.52	0.40	0.00
rs475011	*RREB1*	6	G	0.69 (0.41, 0.98)	5.65 × 10^−6^	0.32 (0.03, 0.61)	0.03305	0.51	1.15 × 10^−6^	0.07
rs76433159	*NELL1*	11	C	0.71 (0.41, 1.01)	8.93 × 10^−6^	-0.31 (-0.61, -0.01)	0.04106	0.19	0.70	0.00
rs72715801	*LPNH2*	1	G	0.99 (0.57, 1.41)	9.16 × 10^−6^	-0.17 (-0.60, 0.16)	0.4451	0.41	0.48	0.00

Beta, Regression coefficient of the tested allele; CI, confidence interval; *p*, *p* value of linear regression; *P*_*combined*_, combined *p* value of discovery and replication stage based on meta-analysis; Q, *p* value for Cochrane’s Q statistic.

a Obtained by random- or fixed-effects model for loci with Q < 0.05 or Q > 0.05, respectively.

The most significantly associated SNP cluster was located at 6p25.1 and its strongest signal came from rs473437 (*p* = 1.4 × 10^−7^). Its minor allele T led to an increase in the T-score of 0.84 (95% CI: 0.54–1.13) per allele in the discovery group. The SNP cluster was located 67 kb upstream of *RREB1*. *RREB1* is a zinc finger transcription factor that binds to RAS-responsive elements in gene promoters. It can upregulate calcitonin (Thiagalingam et al., 1996). SNPs in the *RREB1* gene have been reported to be associated with heel BMD in individuals of European descent (Kemp et al., 2017; Kim, 2018; Morris et al., 2019).

### The Replication Stage of GWAS Verified Associations Between SNPs Upstream of *RREB1* and BMD in PMO Patients

The leading SNPs in each of the 6 BMD-associated SNP clusters and 1 additional SNP in the SNP cluster upstream of *RREB1* (which was the second leading SNP in this cluster; the leading SNP of this cluster had a missing rate of 8.6%, Supplementary Table S2) were selected for validation in the additional 191 PMO patients (Table 1). Using linear regression with age and BMI as covariates, three out of the seven tested SNPs were significantly associated with BMD. These comprised 2 upstream of *RREB1* and 1 from *NELL1* on chromosome 11 (Table 2, *p* < 0.05). After combining the results from the discovery and replication stages in a meta-analysis, only the two SNPs upstream of *RREB1* (rs473437 and rs475011, Table 2) were associated with BMD in PMO patients. These two SNPs exhibit perfect linkage disequilibrium (LD; *r* ^2^ = 1) in Han Chinese individuals, according to the data from the 1000 Genome Project. And rs475911 showed the most significant association signal with minor allele G related to increased T-score (*p* = 1.15 × 10^−6^, beta = 0.51, Table 2).

**FIGURE 3:**
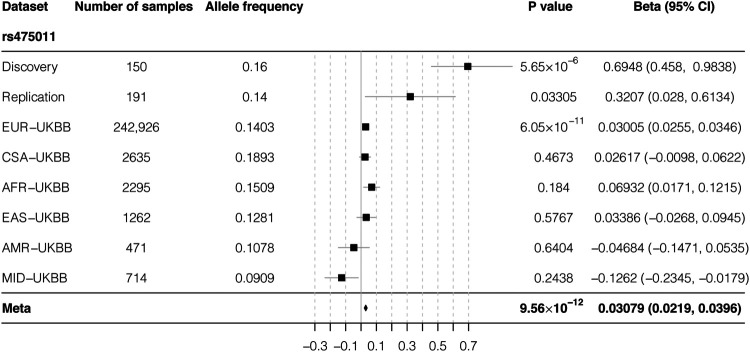
Forest plot of meta-analysis result of rs475011. By combining result from our samples with UKBB summary statistics, a significantly association between rs475011 and T-score was obtained (*p* = 9.56 × 10^−12^, beta = 0.0308). Sample size of each ancestry and analysis group is listed on the left of the figure and the association results are list on the right. X-axis represents the beta value and Y-axis represents the six ancestry groups from UKBB and the discovery and replication group of our study. UKBB ancestry groups are rank by the sample size. The position of the square is the point estimation of the beta value from each group. The diamond represents the combined results. The width of the diamond indicate the 95% confidence interval. UKBB: UK biobank; EUR: European ancestry; CSA: Central/South Asian ancestry; AFR: African ancestry; EAS: East Asian ancestry; AMR: Admixed American ancestry; MID: Middle Eastern ancestry.

As a relatively small sample size was used in our study, we used a publicly available data set to further validate our results. According to data from The Musculoskeletal Knowledge Portal, the A allele of rs475011 is significantly associated with decreased estimated BMD among ∼570,000 samples (*p* = 5.69 × 10^−12^, beta = ^−^0.02) (Kiel et al., 2020). This result is consistent with our finding that the G allele of rs475011 is associated with an increased T-score (Table 2). We also analyzed the relationship between rs475011 and heel BMD T-score using summary statistics data from UKBB. As shown in Figure 3, the G allele of rs475011 was significantly associated with increased T-score in European samples (*p* = 6.05 × 10^−11^, beta = 0.030). Except for samples of Admixed American and Middle Eastern descents, the G allele was related to increased T-score in the remaining populations (Figure 3). By combining all data sets assessed (discovery, replication, and UKBB), the association between rs475011 and T-score reached *p* = 9.56 × 10^−12^, beta = 0.031 (Figure 3). Meta-analysis of our samples with East Asian descents of UKBB also indicated the contribution of the rs475011 G allele to T-score increasing (*p* = 0.0035, beta = 0.153).

### BMD-Associated SNPs May Affect *RREB1* Expression

**FIGURE 4:**
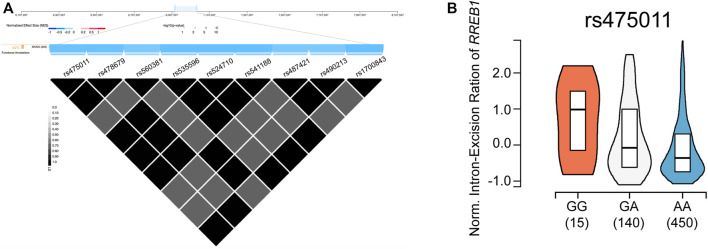
rs475011 acts as a splicing quantitative trait locus (sQTL) of *RREB1*. **(A)** Upper layer: physical position (based on human reference genome hg38) of the SNP cluster containing rs475011. Middle (blue) layer: major alleles of the labeled SNPs are negatively associated with the intron excision ratio of *RREB1*. Lower triangle plot: linkage disequilibrium (D′) of SNPs in this cluster. Data were obtained from GTEx database. **(B)** Allele G of rs475011 is associated with an increased intron excision ratio of *RREB1*. Y-axis represents the normalized intron excision ratio of *RREB1*. The X-axis represents different genotypes of rs475011. Data were obtained from GTEx database.

As the BMD-associated SNP cluster was located 67 kb upstream of *RREB1*, we further checked the regulatory effects of these SNPs by querying the Genotype-Tissue Expression (GTEx) database (Consortium, 2020). As shown in Figure 4A, this region is known to act as a splicing quantitative trait locus (sQTL) of *RREB1* in the skin (Garrido-Martin et al., 2021). The high BMD-associated allele G of rs475011, which was the leading SNP in this cluster in the meta-analysis, was significantly associated with an increased intron excision ratio of *RREB1* (Figure 4B, *p* = 2.1 × 10^−6^). Moreover, this SNP is located in the binding site of two transcription factors, *RFX3* and *FOXB1*, according to chromatin immunoprecipitation (ChIP)-seq data and prediction, respectively, based on information in the RegulomeDB database (Jolma et al., 2010; Boyle et al., 2012; Jolma et al., 2013). This indicates that rs475011 affects BMD in PMO patients by regulating *RREB1* expression.

## Discussion

In this study, we performed a GWAS of BMD in PMO patients and identified SNPs upstream of *RREB1* that significantly influenced BMD. GWAS is a powerful tool for identifying genetic factors related to various phenotypes, and it has been used to identify osteoporosis susceptibility genes in many studies (Yang et al., 2020; Zhu et al., 2021). A GWAS of half a million European participants linked BMD with >500 genetic loci (Morris et al., 2019), but this kind of research on Chinese individuals is still lacking. Although we only included 341 participants, the two-step GWAS design, the replication on UKBB summary statistics, and significant results make our study valuable for understanding the genetic basis of PMO in Chinese individuals.

Sample size is a critical parameter that influences the statistical power of GWAS (Spencer et al., 2009). Based on the GWAS power calculator described by Visscher et al. (Visscher et al., 2017), along with the beta value (0.51) and MAF of rs475011 (0.108) and the sample size of 341, the estimated detection power of our study was 0.98. This further demonstrates the reliability of our results. As genetic research on Chinese PMO is limited, further research involving large samples is highly important for finding genetic loci with small effect sizes (Spencer et al., 2009; Yang et al., 2020).

PMO is a late-onset disease with high prevalence. As it is hard to define appropriate controls (Warming et al., 2002; Chen et al., 2016; Bijelic et al., 2017), we conducted a quantitative trait GWAS to avoid misclassification of controls. The genetic correlation coefficients among various BMD measurements assessed using different platforms (e. g., DXA, qCT, or ultrasound) varies from 0.505 to 0.917 (Karasik et al., 2017). Hence, to reduce the influence of heterogeneity caused by different measurement platforms, we used the T-score (based on standard normalized BMD data measured using different platforms for different subjects), which is a relative value of BMD, as the quantitative trait in the GWAS. Moreover, BMD measured at different skeleton sites may also introduce heterogeneity to the data (Yang et al., 2020). Therefore, we obtained lumbar vertebra BMD values for all participants to avoid this heterogeneity. These measures decreased the confounding factors in our study.

The most significant high BMD-associated SNP in our study, rs475011, was located upstream of *RREB1*. This region is known to act as an sQTL of *RREB1*, according to data from the GTEx database (Figure 3). The high BMD-associated allele G of rs475011 is also linked to an increased intron excision ratio of *RREB1*, based on GTEx data. Higher intron excision indicates upregulation of mature *RREB1* mRNA, suggesting that *RREB1* upregulation may help to prevent bone mineral loss in elderly women. Our results are consistent with previous GWASs of individuals of European descent (Kemp et al., 2017; Kim, 2018), which reported that the minor allele A of rs525678, upstream of *RREB1*, was associated with increased BMD. According to data from the 1000 Genomes Project and GTEx database, in individuals of European descent, rs525678 was in high LD (D’ = 0.998) with rs2842895, which is an expression quantitative trait locus (eQTL) of *RREB1* (Genomes Project et al., 2015). The minor allele C of rs2842895 is linked to *RREB1* upregulation (Consortium, 2020). Moreover, by analyzing data from UKBB, we found the rs475011 is also associated with BMD in individuals of European descent. There is moderate LD between rs475011 and rs2842895 (D’ = 0.719), which indicates that both of the loci contribute to BMD in individuals of European descent. In contrast, rs2842895 is a rare SNP in Han Chinese individuals (MAF = 0.005), which indicates that rs475011 is the major SNP that affects BMD in Han Chinese individuals. Taking the results together, high *RREB1* expression may be a protective factor regarding the development of PMO.

RREB1 is a transcription factor that binds to RAS-responsive elements in gene promoters. The calcitonin gene promoter has a binding site for RREB1, and RREB1 binding upregulates calcitonin (Thiagalingam et al., 1996). Calcitonin is widely used to treat osteoporosis in menopausal women (Reginster, 1993). It may play a protective role regarding BMD by inhibiting the bone resorption function of osteoclasts (Keller et al., 2014). The high BMD-associated alleles identified in both our research (G allele of rs475011) and previous research (A allele of rs525678) may upregulate calcitonin via *RREB1* upregulation (Kemp et al., 2017; Morris et al., 2019). In turn, upregulated calcitonin may lower osteoclast activity and prevent bone mineral loss in postmenopausal women.

To overcome the under statistical power caused by the small sample size of our study, we obtained genome-wide association summary statistics of rs475011 with T-score from UKBB pan-ancestry genetic analysis portal. Although a meta-analysis of all sample groups showed a significant result, among UKBB populations, a significant association was only observed in European descents (*p* = 6.05 × 10^−11^, Figure 3). This might be caused by the relatively small sample size of the non-European populations, and the results in 242,926 European samples may be more robust and trustable. Moreover, UKBB samples were collected from individuals under various health conditions, of which the PMO only accounted for a small proportion (Sudlow et al., 2015). In contrast, our samples were all PMO patients with T-score < −2.5, and rs475011 may contribute to the protective against PMO, which can also be inferred by the role of *RREB1* on regulation calcitonin. The protective role can explain the smaller beta value in the UKBB data in comparison with our data (Figure 3). Additionally, since East Asian descents of UKBB may include limited individuals of PMO, this can explain the absence of the association in them. This speculation was further supported by the fact that the protective effect of the rs475011 G allele was larger in our discovery group than the replication group (beta = 0.69 vs. 0.32, Table 2) since the discovery group had a smaller average T-score or severe PMO condition (*p* = 0.016, t-test, Table 1 and Figure 3).

The results of our study may help to understand PMO in Chinese individuals; however, several limitations should be considered. First, the sample size was relatively small. Although the estimated detection power of our study for loci with large effect sizes (beta >0.5) is 0.98, the sample size is insufficient for identifying loci with small effect sizes, which may act as major genetic contributors of common diseases (Boyle et al., 2017). Second, the information on the relationships of the BMD-associated SNPs with *RREB1* was gleaned from databases. Although our study and previous publications (Kemp et al., 2017; Kim, 2018) obtained concordant results, there is a lack of direct functional evidence linking the reported SNPs with the expression of *RREB1* and calcitonin.

In conclusion, we conducted a GWAS of BMD based on Chinese PMO patients and found that an *RREB1* allele that increases the intron excision ratio is associated with increased BMD. The results are consistent with previous research on individuals of European descent. However, this is a preliminary study, and future association studies should include large numbers of Chinese PMO patients. eQTL, sQTL, and functional analyses of rs475011, *RREB1*, and calcitonin should be carried out.

## Data Availability

The original contributions presented in the study are included in the article/Supplementary Material, further inquiries can be directed to the corresponding author/s.
